# An Optically Tunable THz Modulator Based on Nanostructures of Silicon Substrates

**DOI:** 10.3390/s20082198

**Published:** 2020-04-13

**Authors:** Chen Mo, Jingbo Liu, Dongshan Wei, Honglei Wu, Qiye Wen, Dongxiong Ling

**Affiliations:** 1 School of Electrical Engineering and Intelligentization, Dongguan University of Technology, Dongguan 523808, China; 2172281583@email.szu.edu.cn (C.M.); liujb@dgut.edu.cn (J.L.); dswei@dgut.edu.cn (D.W.); 2 College of Physics and Optoelectronic Engineering, Shenzhen University, Shenzhen 518061, China; hlwu@szu.edu.cn; 3 State Key Laboratory of Electronic Thin Films and Integrated Devices, University of Electronic Science and Technology of China, Chengdu 610054, China; qywen@uestc.edu.cn

**Keywords:** terahertz, modulator, nanostructure, CST simulation

## Abstract

Nanostructures can induce light multireflection, enabling strong light absorption and efficient photocarrier generation. In this work, silicon nanostructures, including nanocylinders, nanotips, and nanoholes, were proposed as all-optical broadband THz modulators. The modulation properties of these modulators were simulated and compared with finite element method calculations. It is interesting to note that the light reflectance values from all nanostructure were greatly suppressed, showing values of 26.22%, 21.04%, and 0.63% for nanocylinder, nanohole, and nanotip structures, respectively, at 2 THz. The calculated results show that under 808 nm illumination light, the best modulation performance is achieved in the nanotip modulator, which displays a modulation depth of 91.63% with a pumping power of 60 mW/mm^2^ at 2 THz. However, under shorter illumination wavelengths, such as 532 nm, the modulation performance for all modulators deteriorates and the best performance is found with the nanohole-based modulator rather than the nanotip-based one. To further clarify the effects of the nanostructure and wavelength on the THz modulation, a graded index layer model was established and the simulation results were explained. This work may provide a further theoretical guide for the design of optically tunable broadband THz modulators.

## 1. Introduction

Terahertz (THz) radiation (0.1~10 THz) triggers a great number of intriguing and complex physical, biological, and chemical phenomena. It consequently possesses wide practical application prospects in communications, spectroscopy, and imaging [1,2,3,4,5,6,7]. For THz imaging purposes, a THz spatial light modulator (SLM) is involved, which requires the THz modulator to be fast and efficient. However, the present THz modulators do not fully meet such requirements, hindering their application in THz imaging

One approach to achieving active modulation of THz radiation is through optically induced modification of device properties. High-resistivity (HR) silicon has been proven to be suitable for optically tunable THz modulations [8]. THz waves can be modulated by optically pumping silicon to form a temporary region with either high absorption or strong reflection [9]. As a result, Okada et al. [10] and Xie et al. [11] proposed silicon-based spatial THz modulators (STM), while Zhang et al. [12] and Cheng et al. [13] reported optically controlled reconfigurable quasi-optical THz devices. Optically tunable THz modulators enable broadband modulation with considerable modulation depth. Nevertheless, the high reflectivity of bare silicon requires high pumping power to achieve adequate modulation depth, which restricts the development of efficient and integrated THz modulators.

Nanostructured surfaces have been proven to form efficient antireflection interfaces [14,15,16,17], and have been widely applied in the field of solar energy. Therefore, it is expected that larger modulation depth can be achieved by a nanostructure-based optical THz modulator in comparison to a bare silicon-based modulator. However, in order to achieve a large modulation depth, a THz modulator based on the silicon nanotip array must have strict restriction conditions, such as the length and fill ratio of the nanostructures [18]. Sometimes high-resistivity silicon with a surface of nanosized pillars has no obvious enhancement effect on modulation depth [19]. Therefore, the mechanism behind the use of nanostructures to enhance the modulation depth of optically tunable THz modulators is not clear. It is necessary to find a theoretical analysis method for optically tunable THz modulators based on nanostructures.

In this paper, in order to carry out a rigorous numerical analysis of optically tunable THz modulators based on nanostructures, nanostructure-based modulators were developed for broadband THz modulation from 0.1 to 4 THz. Meanwhile, the THz modulation depths of these models based on bare silicon, nanocylinders [20,21,22], nanotips [23,24], and nanoholes [25] were simulated and analyzed under different pumping powers and pump beam wavelengths. The results show that the THz modulation depth of nanostructure-based modulators is larger than that of the bare silicon-based modulator. A longer pump beam wavelength leads to a larger THz modulation depth. Moreover, a numerical model was established to further understand the simulation, and the calculated THz reflectance results are in accordance with simulation results.

## 2. Method

**Figure 1:**
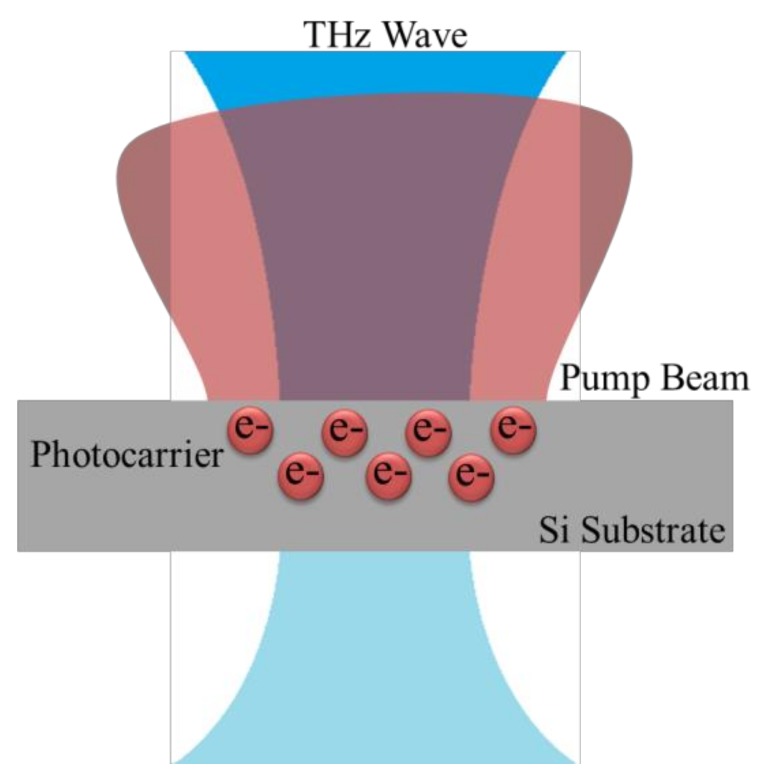
Schematic of the optically tunable THz modulator.

As shown in Figure 1, for the silicon-based optically tunable THz modulator, photocarriers are generated in silicon upon pump beam illumination. The silicon conductivity then increases with the increasing photocarrier concentration, resulting in attenuation of THz transmittance. Thus, amplitude modulation of THz waves is enabled.

The excited photocarrier concentration (*N*) is closely related to the pump beam power loss in the semiconductor, and can be estimated as follows:

(1) $$N=\frac{P}{h\nu V}$$

where *P* is the pump beam power loss in the semiconductor, *h* is the Planck constant, *ν* is the frequency of the pump beam, and *V* is the volume of the semiconductor. Since the concentration of intrinsic carriers is very low in high-resistivity silicon, the excited photocarrier concentration is assumed to be equivalent to the carrier density in the semiconductor. The power absorption of the semiconductor increases with the increase of the pump beam power, leading to a higher photocarrier density value.

The Drude model can be used to describe photocarrier absorption, and the simulated THz wave transmittance is mainly determined by photoconductivity [26]. It can be derived that the plasma frequency (*ω_p_*) depends on the photocarrier density according to the equation:

(2) $$\omega_{p}=\sqrt{\frac{Ne^{2}}{\varepsilon^{*}m^{*}}}$$

where *e* is the electric charge, ε* is the dielectric constant, m* = 0.26 × *m_0_* is the effective electron mass, and *m_0_* is the electron mass. According to the Drude model, the photoconductivity is expressed as follows:

(3) $$\sigma_{Drude}=\frac{\varepsilon_{0}\gamma \omega_{p}^{2}}{\left(\omega^{2}+\gamma^{2}\right)}$$

where *ε_0_* is the vacuum permittivity, *γ* is the relaxation rate, and *ω* is the angular frequency of the THz wave, which is calculated by 2 THz (the middle of 0.1~4 THz). The relaxation rate *γ* is set to 5 × 10^13^ s^−1^ considering that the estimated photocarrier concentration is about 10^16^~10^18^ cm^−3^ [27,28]. Regarding Equations (1)–(3), photoconductivity (*σ_Drude_*) increases with the increasing pump beam power, which further influences THz wave transmittance, and thereby, the modulation depth.

Periodic arrays of three different nanostructures, namely nanocylinders, nanotips, and nanoholes, were designed to explore the corresponding THz modulation properties. The THz modulation properties based on bare silicon were also calculated for comparison. Computer Simulation Technology Microwave Studio (CST MWS) was employed for the simulation.

The MW & RF & OPTICAL modules of CST MWS were used to simulate the pump beam power loss in the semiconductor and the THz wave modulation properties of the periodic nanostructure arrays. Two simulations needed to be performed. The first simulation in the time domain solver was to performed to acquire the pump beam power loss in the semiconductor. Periodic boundary conditions were employed to simulate the periodic nanostructures on a unit cell in the *x*- and *y*-directions, respectively. In the z-direction, waveguide ports were set to emit and detect light waves with open boundary conditions applied. This setting ensures the normal incidence of the pump beam at the surface of the structure. The wavelength and amplitude of the pump beam could be set up as excitation signals. The pump beam power could be tuned by selecting the source type in the solver setup. A series of pump beam power densities ranging from 5 to 60 mW/mm^2^ with a step of 5 mW/mm^2^ were applied in simulation. The associated photoconductivity values of the nanostructures could then be estimated. The other simulation was performed to simulate THz wave modulation with different photoconductivity values. Unit cell boundary conditions were employed to simulate the nanostructures in the *x*- and *y*-directions, respectively, while the frequency domain solver was applied to simulate frequency-dependent THz modulation at 0.1-4 THz. The polarization direction of the pump beam and THz wave was in the y-direction. The silicon substrate studied in the simulations was polycrystalline silicon. The nanostructure model was symmetric in x- and y-directions and had the same periodic conditions. In this simulation, the influence of temperature rise caused by illumination is not considered.

**Figure 2:**
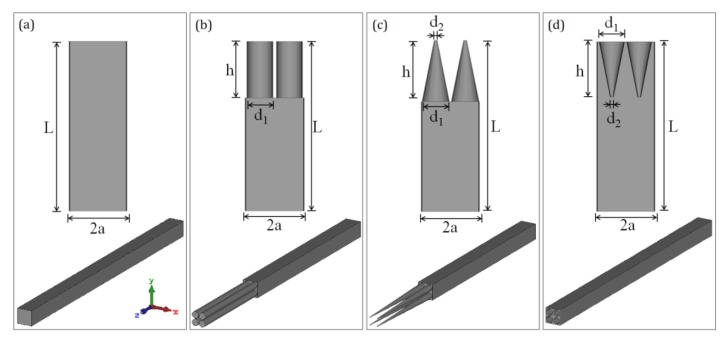
The dimensional distributions and 3D models of different nanostructures in the simulation: (**a**) bare silicon, L = 15 μm, a = 400 nm; (**b**) nanocylinders on the Si substrate, h = 5 μm, d_1_ = 350 nm, L = 15 μm, a = 400 nm; (**c**) nanotips on the Si substrate, h = 5 μm, d_1_ = 350 nm, d_2_ = 40 nm, L = 15 μm, a = 400 nm; (**d**) nanoholes on the Si substrate, h = 5 μm, d_1_ = 350 nm, d_2_ = 40 nm, L = 15 μm, a = 400 nm.

Afterwards, four types of Si nanostructures were devised, as shown in Figure 2. The bare silicon was 15 μm in height. A 10-μm thick silicon substrate was applied for the nanocylinder array (350 nm in diameter and 5 μm in height) and nanotip array (40 nm in top diameter, 350 nm in base diameter and 5 μm in height), while nanoholes measuring 40 nm in base diameter, 350 nm in top diameter, and 5 μm in depth were arranged on a 15-μm thick silicon substrate to form the nanohole array. The period for these four models described above was 400 nm. The nanocylinder and nanotip structures were designed based on silicon nanowires (SiNW) [29] and silicon nanotips (SiNT) [18] reported in previous studies. The nanohole structure was devised as a comparison with the nanotip structure, while the bare silicon was set as a reference for all three nanostructure arrays. Considering the Bragg condition (λ~2a), 2 × 2 arrays were employed on a unit cell to avoid light diffraction at the pump beam wavelengths under 808 nm, as shown in Figure 2 [30].

## 3. Results and Discussion

### 3.1. Simulation Results

**Figure 3:**
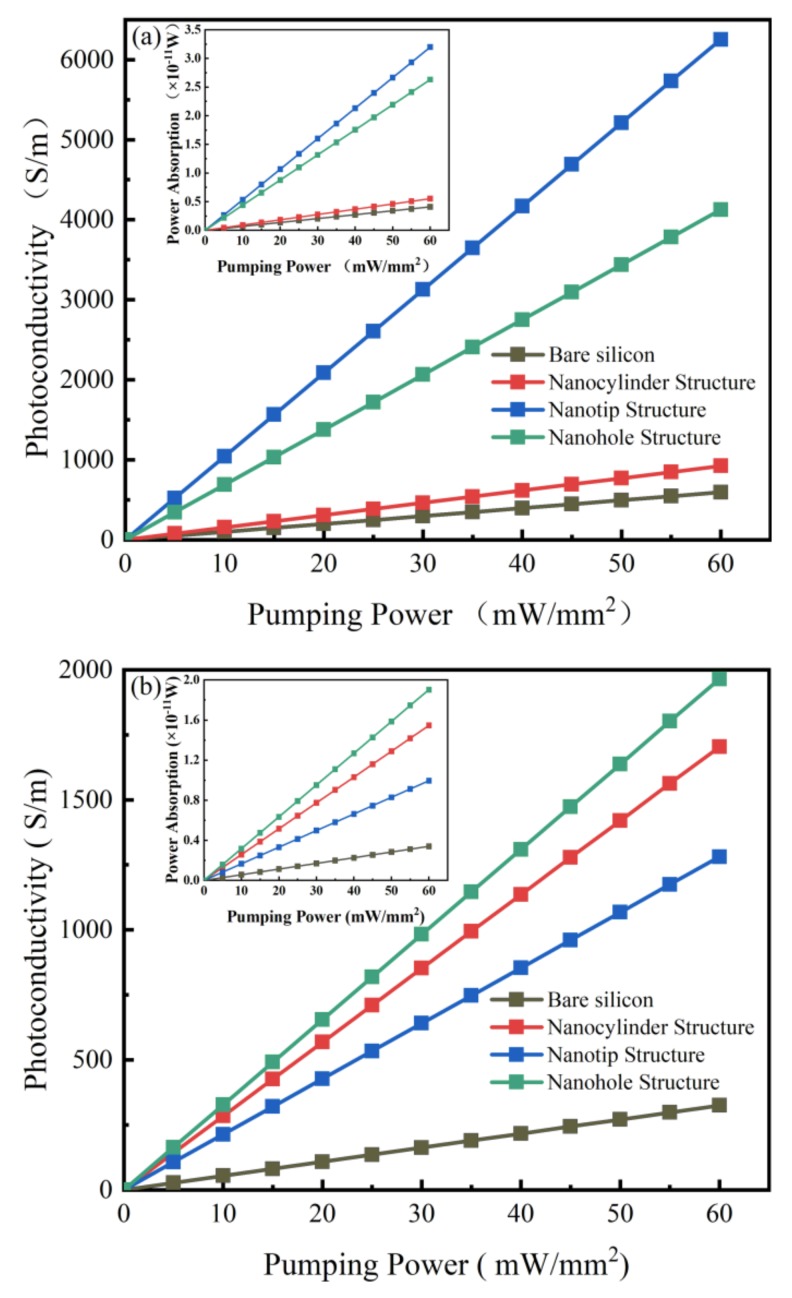
The power absorption (inset) and photoconductivity values of the bare silicon, nanocylinder, nanotip, and nanohole structures versus pumping power densities with a (**a**) 808 and (**b**) 532 nm pump beams, respectively.

Common pump beams with wavelengths of 808 and 532 nm [18,31,32,33,34], respectively, and silicon resistivity of 1000 Ω∙cm were employed to simulate the THz modulation with different nanostructures. As is known, longer wavelengths have a stronger ability to penetrate silicon. Therefore, the excitation of 808 nm light enables bulk excitation of silicon, while the excitation of 532 nm light enables only surface excitation. In our work, we mainly study the influence of the nanoscale structure of the silicon surface on its excitation. The research area scale is small, only including the area relatively close to the surface. Therefore, the excitation of 532 nm light can be considered to be bulk excitation in the area. The photoconductivity values of bare silicon, nanocylinder, nanotip, and nanohole structures versus pumping power densities are shown in Figure 3, with the inset presenting the power absorption variation. The photoconductivity value was calculated from the power absorption according to Equations (1)–(3), and the power absorption was obtained from the first simulation.

As shown in the inset of Figure 3a, the power absorption was found to be in a linear relationship with pumping power for all the models. However, the nanotip, nanohole, and nanocylinder structures display a power absorption values of 7.8, 6.3, and 1.3 times higher than that of the bare silicon under the same conditions, respectively. For example, under the same pumping power of 60 mW/mm^2^, the nanotip structure exhibits the highest power absorption (3.2 × 10^−11^ W) among the four models, followed by the nanohole structure with a power absorption of 2.6 × 10^−11^ W. The power absorption values of the nanocylinder structure and bare silicon are much lower, being 5.5 × 10^−12^ W and 4.1 × 10^−12^ W, respectively. The sharp contrast indicates that the nanotip and nanohole structures have higher absorption efficiency with the 808 nm pump beam compared to the nanocylinder structure and bare silicon.

The photoconductivity was also found to be positively and linearly related to the pumping power with the 808 nm pump beam, as shown in Figure 3a. Under the same pumping power, the photoconductivity values of nanotip, nanohole, and nanocylinder structures are 10.5, 6.9, and 1.6 times higher than that of bare silicon, respectively (e.g., with a pumping power of 60 mW/mm^2^, the photoconductivity values of the nanotip, nanohole, and nanocylinder structures and bare silicon are 6253, 4126, 923, and 594 S/m, respectively).

The significantly improved properties of the nanotip array are due to the enhanced multireflection among the nanotips and the specific surface area, which enables strong light absorption and photocarrier generation, thereby resulting in a significant increase in the photoconductivity. Enhanced light multireflection also occurs for the nanohole array, but unfortunately this only happens for light that falls into the holes, while the rest of the light is reflected from the surface. Therefore, the photoconductivity of the nanohole array is slightly lower than that of the nanotip array. As for the nanocylinder array, most of the light is reflected from the cylinder surface and only the diffraction light takes part in the multireflection, resulting in much lower photoconductivity. In contrast, the lowest photoconductivity is observed for bare silicon due to the absence of light multireflection.

A pump beam with a wavelength of 532 nm was also simulated to investigate the relationship between the THz modulation depth and the pump beam wavelength. As shown in the inset of Figure 3b, linear relationships similar to those obtained with the 808 nm pump beam were again observed between the power absorption and pumping power for all models. Nevertheless, the difference is that under the same pumping power with the 532 nm pump beam, the power absorption of the nanotip structure was lower than for the nanohole and nanocylinder structures. Additionally, the power absorption of the nanocylinder structure was higher with the 532 nm wavelength than with the 808 nm wavelength. For instance, with the 60 mW/mm^2^ pumping power, the corresponding power absorptions of bare silicon, nanocylinder, nanotip, and nanohole structures were 3.4 × 10^−12^ W, 1.5 × 10^−11^ W, 9.9 × 10^−12^ W, and 1.9 × 10^−11^ W, respectively. As will be described below, these behaviors are caused by Bragg diffraction. As for photoconductivity, the values for all these models with the 532 nm pump beam also show a similar upward variation trend with power absorption, as shown in Figure 3b. Under the same pumping power, the photoconductivity values of the nanohole, nanocylinder, and nanotip structures are 6, 5.2, and 3.9 times higher, respectively, than that of bare silicon, with the corresponding specific values being 1965, 1704, 1281, and 325 S/m at 60 mW/mm^2^. The photoconductivity values of the four models at 60 mW/mm^2^ are summarized in Table 1.

As mentioned above, different simulation results were obtained for pump beams with 532 and 808 nm wavelengths. The differences are explained by the observation that the photoconductivity of the nanotip array with the 532 nm pump beam was lower than those of the nanohole and nanocylinder structures. This is because that the top diameter and the bottom diameter of the nanotip structure are 40 nm and 350 nm, respectively. The diameter of the nanotip structure between the top and the bottom region will be in accord with the half wavelength of 532 nm. Strong Bragg diffraction occurred for the nanotip array as part of the structure dimension fulfilling the Bragg condition [30], resulting in lower absorption of the pump beam, and thereby reduced photoconductivity and modulation depth. Similarly, the lower photoconductivity of the nanocylinder array with the 808 nm pump beam is also because the dimensions of the nanocylinder structure (350 nm in diameter) meet the Bragg condition, and the consequent Bragg diffraction leads to low modulation efficiency for the nanocylinder structure with the 808 nm pump beam, showing only slightly higher results than that of bare silicon.

The photocarrier concentrations of the four models at 60 mW/mm^2^ are listed in Table 2, which are calculated from Equation (1). The enhanced multireflection between the surface nanostructures and the specific surface area enables strong light absorption. According to Equation (1), strong light absorption results in high photocarrier concentration. With the 808 nm pump beam, the nanotip structure achieves the highest photocarrier concentration of 18.25 × 10^24^ m^−3^ among the four models at 60 mW/mm^2^, while the nanohole structure generates the highest photocarrier concentration of 5.74 × 10^24^ m^−3^ with the 532 nm pump beam. The differences in the photocarrier concentrations among the four models at different conditions are explained above.

**Figure 4:**
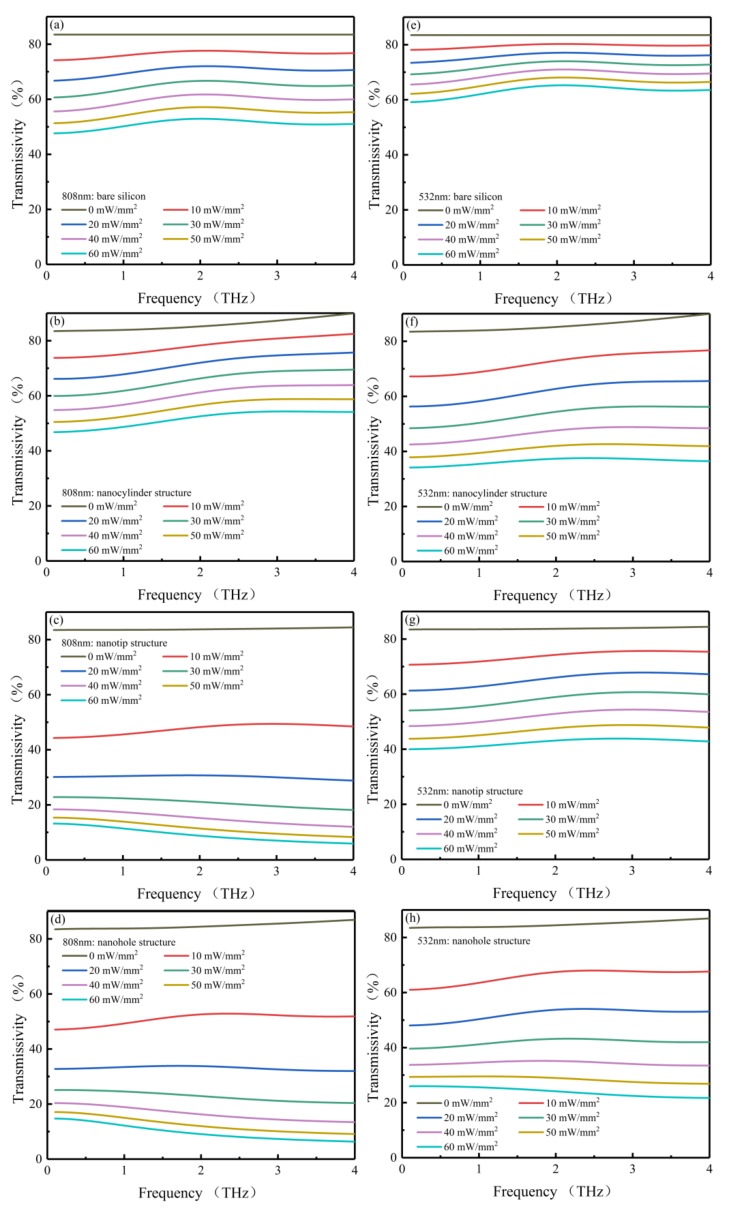
The frequency-resolved transmissivity values (0.1~4 THz) of bare silicon, nanocylinder, nanotip, and nanohole structures with 808 (**a**–**d**) and 532 nm (**e**–**h**) pump beams, respectively.

**Figure 5:**
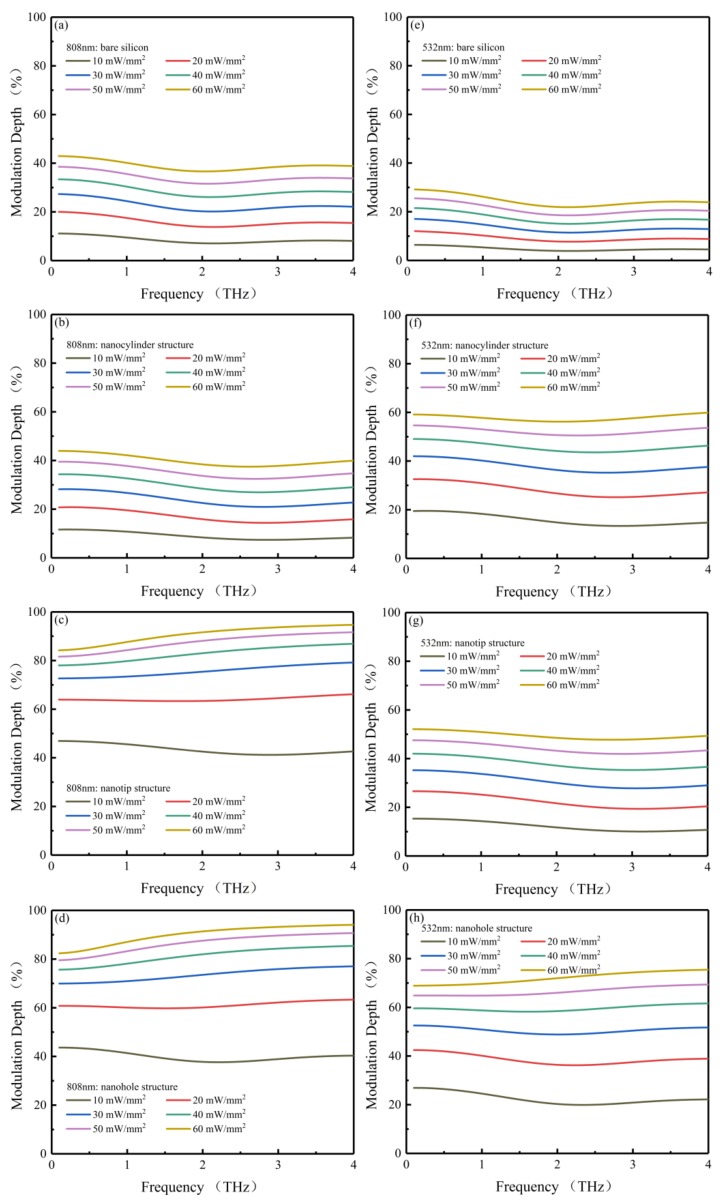
The frequency-resolved modulation depths (0.1~4 THz) of bare silicon, nanocylinder, nanotip, and nanohole structures with 808 (**a**–**d**) and 532 nm (**e**–**h**) pump beams, respectively.

As shown in Figure 4 and Figure 5, with increasing pumping power, the THz transmissivity decreases and the THz modulation depth increases in all four models. Considering that the concentration of intrinsic carriers in high-resistivity silicon (1000 Ω∙cm) is far lower than that of photocarriers, the absorption of THz radiation by intrinsic carriers can be ignored in the simulations. Figure 4 shows the transmissivities of bare silicon, nanocylinder, nanotip and nanohole structures with the pump beam wavelengths of 808 (Figure 4a–d) and 532 nm (Figure 4e–h), respectively, the broadband frequency region ranging from 0.1 to 4 THz. As shown in this figure, in the absence of a pump beam, the frequency-averaged transmissivity values of bare silicon, nanocylinder, nanohole, and nanotip structures are almost equivalent at 83.47%, 85.78%, 84.75%, and 83.82%, respectively. Upon applying the 808 nm pumping power of 60 mW/mm^2^, the frequency-averaged transmissivity values of nanotip and nanohole structures dropped to 9.13% and 9.69%, respectively, while reduced frequency-averaged transmissivity values were also observed for the nanocylinder structure and bare silicon, resulting in values of 51.63% and 51%, respectively (Figure 4a–d). As for the simulation with the 532 nm pump beam (Figure 4e–h), under a pumping power of 60 mW/mm^2^, the frequency-averaged transmissivity values of nanohole, nanocylinder, and nanotip structures and bare silicon were found to decrease to 23.96%, 36.41%, 42.44%, and 63.18%, respectively. The frequency-averaged transmissivities of the four models at 60 mW/mm^2^ are summarized in Table 3.

When silicon is under illumination, one electron absorbs a photon and moves to the excited state, producing photoexcited electrons and holes—so-called photocarriers. The production of photocarriers alters the conductivity of silicon. A higher pumping power generates a larger number of photocarriers, and thus a higher photocarrier concentration, which enhances the conductivity of silicon. Consequently, more THz waves will be absorbed and reflected, resulting in lower THz transmittance, and thereby larger THz modulation depth.

In this work, the modulation depth is defined as $\mathrm{MD}=\left(T_{0}-T_{p}\right)/T_{0}\;$, where *T_0_* is the THz transmittance in the absence of a pump beam and *T_p_* is the THz transmittance under various pumping powers.

As can be seen from Figure 5a–d, in the broadband frequency region ranging from 0.1 to 4 THz, the nanotip structure presents good modulation effects with the 808 nm pump beam. Even under a low pumping power of 10 mW/mm^2^, a frequency-averaged modulation depth of 43.41% was achieved in the nanotip structure; in comparison, the frequency-averaged modulation depths of bare silicon, nanocylinder, and nanohole structures were 8.48%, 9.07%, and 39.93%, respectively. The modulation depth of the nanotip structure was five times that of bare silicon, confirming that effective modulation of THz waves can be achieved by the nanotip structure. After tuning the pump power density up to 60 mW/mm^2^, the frequency-averaged modulation depth of the nanotip and nanohole structures reached up to 90.74% and 90.14%, respectively, while the values of bare silicon and the nanocylinder structure were 38.99% and 39.94%, respectively. The large modulation depth implies that almost all THz waves are blocked in the nanotip and nanohole structures, in accordance with Figure 4c–d. Therefore, with the 808 nm pump beam, the nanotip and nanohole structures are much more efficient in optically tunable THz modulation.

From the above information, It can be derived that higher pumping power absorption leads to increased photocarrier concentration, and thus a larger THz modulation depth. Among the four models, the nanotip structure has the highest absorption with the 808 nm pump beam, followed by the nanohole structure, nanocylinder structure, and bare silicon. Due to the connection between the power absorption and modulation depth, the THz modulation efficiency of these models follows exactly the same order.

It is also noteworthy that bare silicon and the nanotip structure achieved modulation depths of 15.5% (15 mW/mm^2^) and 38.2% (50 mW/mm^2^), and 56.9% (15 mW/mm^2^) and 82% (50 mW/mm^2^) at 0.34 THz, respectively, as obtained from the simulation. These values are in good agreement with the experiment results [18] under the same conditions (808 nm pump beam and 1000 Ω∙cm silicon).

The simulated modulation depth results with the 532 nm pump beam are shown in Figure 5e–h. Under a low pumping power of 10 mW/mm^2^, the frequency-averaged modulation depths of the nanohole, nanocylinder, and nanotip structures and bare silicon approach 22.47%, 15.8%, 12.19%, and 4.76%, respectively; under the60 mW/mm^2^ pumping power, the modulation depths of these models reach up to 72.1%, 57.7%, 49.42%, and 24.47%, respectively. Only the nanohole structure can acquire adequate modulation depth with the 532 nm pump beam. This illustrates the lower modulation efficiency with the 532 nm pump beam in comparison to the 808 nm pump beam. Similar results have also been reported in experiments that compared the difference in modulation efficiency between 450 and 800 nm pump beams [26]. The frequency-averaged modulation depth of the four models from 10 to 60 mW/mm^2^ are summarized in Table 4.

The THz modulation depth differences between the pump beams with different wavelengths can be quantitatively explained by two aspects: (1) The relationship between the light wavelength and photon number. It is well-known that longer pump beam wavelengths can provide more photons under the same pumping power. Photon energy can be obtained using the equation *E[eV] = hν/1eV*, where *h* is the Planck constant and *ν* is the frequency of the pump beam. The ratio of photon numbers contained in 808 (1.53 eV) and 532 nm (2.33 eV) pump beams under the same pumping power is 2.33/1.53 ≈ 1.52, indicating that compared to the 532 nm pump beam, more photocarriers are generated under the 808 nm pump beam due to the presence of more photons. (2) The reflectivity of silicon varies under different wavelengths. The reflectivity values of silicon for 532 and 808 nm lights are 0.38 and 0.33, respectively [35,36]. Clearly, given the same incident photon numbers, more photons will be reflected by silicon with the 532 nm wavelength compared to photons with the 808 nm wavelength. Therefore, under the same pumping power, the 808 nm pump beam contains more photons and may have more photons penetrating the silicon.

**Figure 6:**
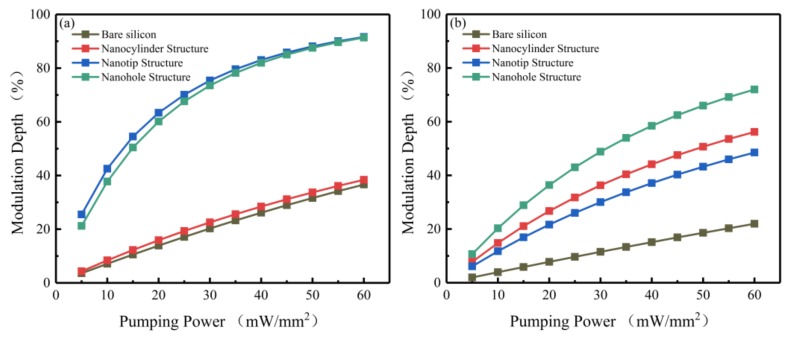
The modulation depths of bare silicon, nanocylinder, nanotip, and nanohole structures at 2 THz with 808 (**a**) and 532 nm (**b**) pump beams as a function of the pumping power.

Figure 6a shows the dependence of the modulation depth on the 808 nm pumping power for the four models at 2 THz. Increasing the pumping power from 5 to 60 mW/mm^2^, the modulation depths of bare silicon and the nanocylinder structure increase in an almost linear manner from 3.6% and 4.32% to 36.65% and 38.42%, respectively. As for the nanotip and nanohole structures, the modulation depths increase rapidly from 25.5% and 21.21% to 91.63% and 91.37%, respectively. The fast saturation is caused by the rapid increase of the photoconductivity of the nanostructures, along with the pumping power (see Figure 3). Silicon blocks almost 80% of the THz wave with a photoconductivity of 3000 S/m, which is achieved in the nanotip and nanohole structures under 30 and 45 mW/mm^2^, respectively (Figure 3a). Although the photoconductivity is positively correlated with the increase of the pumping power, the corresponding wave transmissivity decreases at a slower speed. Therefore, the growth rate of the modulation depth decreases quickly with the increase of pumping power. This phenomenon can also be explained by Pauli blocking as a consequence of Pauli exclusion in doped semiconductors [26,37]. Due to Pauli blocking, the phase space available for electron transition is gradually reduced, thus restricting the increase of photocarrier concentration [38,39]. Additionally, the reduced photocarrier lifetime under high pumping power also contributes to this behavior [40]. In this case, the photocarrier concentration tends to be saturated with further increase of the pumping power, resulting in saturated modulation.

The modulation depths of the four models versus the 532 nm pumping power at 2 THz are shown in Figure 6b. As the pumping power increases from 5 to 60 mW/mm^2^, the modulation depth of bare silicon linearly increases from 1.97% to 21.96%. The modulation depths of the nanohole, nanocylinder, and nanotip structures increase from 10.68%, 7.78%, and 6.12% to 71.98%, 56.19%, and 48.54%, respectively. Obviously, none of four models reaches the saturation modulation effect due to the low modulation efficiency with the 532 nm pump beam.

According to the results and discussion above, higher pump beam absorption will enhance the photoconductivity, resulting in larger THz modulation depth. The enhanced multireflection between the surface nanostructures and the specific surface area enables strong light absorption and photocarrier generation. The increased photocarriers enhance the Drude absorption of the THz wave, resulting in significant improvement of the THz modulation depth. Under the 808 nm illumination light, the best modulation performance is achieved with the nanotip-based modulator, while under shorter illumination wavelengths, such as 532 nm, the best performance is found with the nanohole-based modulator. Therefore, nanostructures with higher absorption achieve larger THz modulation depths than bare silicon under the same conditions. Furthermore, compared with the 532 nm pump beam, larger THz modulation depth can be achieved with the 808 nm pump beam.

### 3.2. Theoretical Model

**Figure 7:**
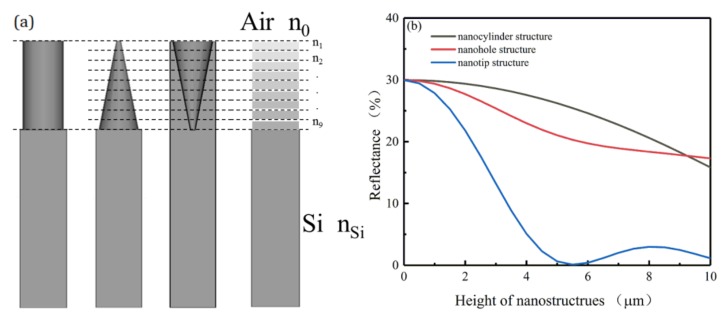
(**a**) The graded index layer model of nanocylinder, nanotip, and nanohole structures. (**b**) The calculated reflectance of the THz waves (2 THz) of nanostructures with different heights.

In order to further understand the influence of nanostructures on THz waves, a graded index layer model [41] was established to explain the simulation results obtained above. This model mainly studies the antireflection effect of nanostructures on THz waves, so the pump beam excitation is not considered. In this model, the nanostructures were regarded as sliced films. Refractive indices of these nanostructures change continuously along the depth, as indicated in Figure 7a. Although more slices may give higher precision, a nine-layer model was used to simplify the calculation.

In order to acquire the reflectance of the nanotip and nanohole structures, the refractive indices were first calculated. For the nanotip structure, the depth-dependent refractive indices of the sliced layers can be described by [42]:

(4) $$n_{k}={\left(1-\frac{n_{Si}-n_{0}}{n_{Si}}\frac{x}{d}\right)}^{-1}$$

where *k* is the number of the sliced layer; *n_0_* and *n_Si_* are the refractive indices of air and Si, respectively; *x = (k/10) × d* represents the depth of the layer; and *d* is the total thickness of the nanostructure. The value of *k* is between 1 and 9 (*k* = 1, 2, …, 9). As for the nanohole structure, the depth-dependent refractive index of the sliced layers can be described by [25]:

(5) $$\alpha_{k}=1-\frac{\pi }{4}\times {\left(1-\frac{x}{d}\right)}^{2}$$

(6) $$n_{k}=\sqrt{\left(1-\alpha_{k}\right)n_{0}^{2}+\alpha_{k}n_{Si}^{2}}$$

where *α_k_* is the filling factor (area fraction) of silicon in each layer. Then, the reflectance values of these two nanostructures were calculated by the transfer matrix method, as follows [43,44]:

(7) $$\left[\begin{array}{c} B\\ C \end{array}\right]=\left(\prod_{K}\left[\begin{array}{cc} \cos\delta_{k} & j\sin\delta_{k}/n_{k}\\ jn_{k}\sin\delta_{k} & \cos\delta_{k} \end{array}\right]\right)\left[\begin{array}{c} 1\\ n_{Si} \end{array}\right]$$

(8) $$R=\left(\frac{n_{0}B-C}{n_{0}B+C}\right)\cdot {\left(\frac{n_{0}B-C}{n_{0}B+C}\right)}^{*}$$

where *B* and *C* are the intermediate variables; $\delta_{k}=\frac{2\pi \eta_{k}x}{\lambda }$, $\eta_{k}=c\sqrt{\frac{\mu \sigma_{k}}{2\omega }}$, $j^{2}=-1$; *c* represents the velocity of light in vacuum; *μ* is the relative permeability of silicon; *σ_k_* represents the conductivity of each layer, which linearly increases from 0 to 0.1 S∙m^−1^ (the resistivity of Si is 1000 Ω∙cm); *λ* and *ω* are the wavelength and the angle frequency of the incident THz wave (2 THz), respectively.

The nanocylinder structure can be represented by one layer model, the THz reflectance of which can be calculated by [42]:

(9) $$R=\frac{{\left(n_{0}-n_{Si}\right)}^{2}\cos^{2}\delta +{\left(\frac{n_{0}n_{Si}}{n_{S}}-n_{s}\right)}^{2}\sin^{2}\delta }{{\left(n_{0}+n_{Si}\right)}^{2}\cos^{2}\delta +{\left(\frac{n_{0}n_{Si}}{n_{S}}+n_{s}\right)}^{2}\sin^{2}\delta }$$

where $\delta =\frac{2\pi n_{s}d}{\lambda }$ is the phase difference between two adjacent layers and *n_s_* is the efficient refractive index. Solving Equations (7)–(9), the reflectance of these nanostructures can be calculated by employing the MATLAB software. This simple theoretical model does not take into account the transfer matrix of the propagation of the THz radiation inside the modulator or the reflection from the second surface. Since the concentration of free carriers in high-resistivity silicon (1000 Ω∙cm) is very low, the absorption of THz radiation by free carriers can be ignored in this model.

The calculated reflectance of nanostructures is shown in Figure 7b. The calculated reflectance of the bare silicon at 2 THz is 29.98%, while that of the nanotip structure with a height of 5.5 μm is 0.1%, representing the lowest reflectance. As for the nanohole and nanocylinder structures, the calculated reflectance decreases as the height increases from 0 to 10 μm. With a height below 9.5 μm, the nanohole structure displays lower calculated reflectance compared with the nanocylinder structure. As illustrated in Figure 7b, the nanostructures can effectively reduce the reflectance of THz waves, with the nanotip structure showing the best antireflection effect.

The calculation results based on the graded index layer model further explain the varied modulation effects simulated for optically tunable THz modulators with different nanostructures. Due to lower insertion loss, nanostructures with lower THz reflectance acquire higher THz modulation depths. With a height of 5 μm, the nanotip structure has the lowest THz reflectance (0.63%), followed by the nanohole structure (21.04%) and nanocylinder structure (26.22%). Correspondingly, under the 808 nm pump beam, the THz modulation depths of the nanostructures follow the sequence: nanotip > nanohole > nanocylinder.

## 4. Conclusions

In summary, the THz modulation depths of bare silicon, nanocylinder, nanotip, and nanohole structures in the broadband frequency region ranging from 0.1 to 4 THz were studied via numerical simulations. Simulation results show that with the 808 nm pump beam, the nanotip structure displays the highest THz modulation depth among the four models, followed by the nanohole structure, nanocylinder structure, and bare silicon. The modulation depth of the nanotip structure is 25.5% at 2 THz under a low pumping power of 5 mW/mm^2^, and can increase to 91.63% upon tuning the pumping power to 60 mW/mm^2^, presenting the saturation modulation effect. Furthermore, according to the simulation results with 532 and 808 nm pump beams, larger THz modulation depths can be achieved with longer pump beam wavelengths. The modulation depth of the nanohole structure at 2 THz increases from 10.68% to 71.98% with the increase of the 532 nm pumping power from 5 to 60 mW/mm^2^. Finally, a graded index layer model was established to understand the influences of nanostructures on the reflectance of the THz wave. At 2 THz, the calculated reflectance values for nanocylinder, nanohole, and nanotip structures with a height of 5 μm were 26.22%, 21.04%, and 0.63%, respectively, confirming the simulation results. We believe that our study provides a theoretical guide for the design of THz modulators, especially for optically tunable broadband THz modulators and micro-nano structures on silicon surfaces.

## Figures and Tables

**Table 1:** The photoconductivity values (S/m) of the four models at 60 mW/mm^2^.

Wavelength	Bare Silicon	Nanocylinder Structure	Nanotip Structure	Nanohole Structure
808 nm	594	923	6253	4126
532 nm	375	1704	1281	1965

**Table 2:** The photocarrier concentrations (×10^24^·m^−3^) of the four models at 60 mW/mm^2^.

Wavelength	Bare Silicon	Nanocylinder Structure	Nanotip Structure	Nanohole Structure
808 nm	1.73	2.70	18.25	12.05
532 nm	0.95	4.97	3.74	5.74

**Table 3:** The frequency-averaged transmissivity values of the four models at 60 mW/mm^2.^

Wavelength	Bare Silicon	Nanocylinder Structure	Nanotip Structure	Nanohole Structure
808 nm	51%	51.63%	9.13%	9.69%
532 nm	63.18%	36.41%	42.44%	23.96%

**Table 4:** The frequency-averaged modulation depths of the four models from 10 to 60 mW/mm^2.^

Wavelength	Bare Silicon	Nanocylinder Structure	Nanotip Structure	Nanohole Structure
808 nm	8.48% → 38.99%	9.07% → 39.94%	43.41% → 90.74%	39.93% → 90.14%
532 nm	4.76% → 24.47%	15.8% → 57.7%	12.19% → 49.42%	22.47% → 72.1%
